# Wicked Problems in Health Professions Education: Adaptive Action in Action

**DOI:** 10.15694/mep.2019.000226.1

**Published:** 2019-12-10

**Authors:** Glenda H. Eoyang, Stewart Mennin

**Affiliations:** 1 Human Systems Dynamics Institute; 2 Human Systems Dyndamics Institute and Mennin Consulting & Associates

**Keywords:** Health Professions Education, Wicked Problems, Pattern, Adaptive Action, Human Systems Dynamics, Complex Adaptive Systems, change

## Abstract

This article was migrated. The article was marked as recommended.

Each educational institution is different. Every clinical setting is unique. Faculty members - clinical, academic, volunteer - are separated by discipline, history, and professional focus. Students begin at different places and learn in different ways. And yet, we focus on the standardization, rigor, and accountability to competencies and curricula in every facet of health professions’ education. The search for consistency and control in such a highly variable system leads inevitably to intractable, wicked problems. Wicked problems are ones that cannot be solved using traditional methods. They are different from traditional problems, in that they are 1) defined differently from multiple perspectives, 2) appear differently in each different context, but follow consistent patterns wherever they appear, and 3) can never be completely solved. Problems that persist for educationalists in health professions meet these criteria and can be classified as “wicked” problems. Rather than solutions to wicked problems, practitioners must choose contextualized, iterative, incremental actions to influence their intractable patterns over time.

## Introduction

At the Association for Medical Education in Europe (AMEE) conference in Helsinki, Finland in 2017, and Basel, Switzerland in 2018 approximately 150 and 100 health professions educators, respectively, engaged in an emergent dialogue process to address the wicked problems they face in their institutions. Using Open Space Technology ( Owen 2008; 2018), the design of the sessions applied principles and practices from human systems dynamics. The purpose was to empower participants and encourage them to plan action that could influence patterns of intractable issues, even when complete solutions were impossible. This paper outlines the intractable problems they identified, explains the shared nature of these diverse issues in terms of complex adaptive systems, shares the principles and practices that were applied during the session, identifies some of the options for action they developed, and proposes recommendations for future research and practice to expand the use of complex adaptive systems theory and human systems dynamics practice to address wicked problems in health professions education.

## The Problem

Some challenges in health professions education are easy to identify, analyze, and solve, but that is not always the case ( Hall, 2002; Huwendiek *et al*., 2010; McKenna, 2012; Schuwirth and Ash, 2013). Other problems persist in spite of investments and innovations ( Conklin, Basadur, and VanPatter, 2007a, 2007b; Irby, 2011; Maniate, 2017; Mennin, Curran, and Eoyang, 2016; Sullivan and Rosin, 2008; Writer, 2015). Table 1 provides a list of such issues that was generated by participants in symposia at the AMEE conferences. Participants were health professions educationalists from a variety of disciplines, roles, and institutions around the world. In spite of their diversity, they shared many of these same intractable issues. Some of the issues were related to curriculum, others to collaboration and conflict. The most frequently named concern was “resistance to change.” This list of issues represents a whole class of challenges faced by the people and institutions engaged in health professions education ( Bleakley, Bligh, and Browne, 2011; Bloom, 1989; Mennin and Krackov, 1998; Mennin, Eoyang, and Nations, 2018).

While these broadly stated issues are consistently named across the wide range of participants, no two situations are identical. Each educational institution is different. Every clinical setting is unique. Faculty members-clinical, academic, volunteer-are separated by discipline, history, and professional focus. Students begin at different places and learn in different ways. And yet, we focus on the standardization, rigor, and accountability to competencies and curricula in every facet of health professions education. The search for consistency and control in such a highly variable system leads inevitably to intractable, wicked problems. Wicked problems are ones that cannot be solved using traditional methods.

**Table 1.** Wicked Problems Identified by Participants at AMEE 2017 and 2018 Symposia

Collaborative planning and implementation of curriculum Competition among roles and values of student, teacher, researcher, clinician Conflict between individuals and among groups Conflict of interest between student, physician, faculty (pass, learn, become a doctor) Creating learning environments that are staged and structured, as well as adapting to individual learning needs and pace Developing clinical practitioners into instructors Developing excellence in volunteer and clinical faculty Developing professional levels of oral and written communication skills Developing self-direction in students Educational inequities in access to technology, insufficient early education, transportation, etc. Faculty resistant to learning about learning, pedagogy, and instruction	Finding trainees who are prepared and motivated High failure rate in first year Implementing new technologies and strategies to instruction Managing at the nexus of teaching and research-finding synergy rather than competition Medical education in time-poor clinical situations Negotiating research agreements across national, institutional, and departmental boundaries Politics in a medical university Promoting interprofessional education Recruitment and retention of general practitioners and family practice physicians Resistance to change Teaching and assessing complex competencies such as ethics and social accountability Teaching and learning “on the job” for junior staff

They do not follow the assumptions required for usual analysis and resolution methods. They emerge from the dynamics of complex adaptive systems, so they exhibit open boundaries, multiple factors, and nonlinear causality ( Cilliers, 1998; Eoyang and Holladay, 2013). All of these characteristics make reductionist, standardized analysis, replication of solutions, and predictable outcomes impossible. Rather than predicting the future, applying best practices, and searching for definitive solutions to such challenges, practitioners must find another path. As we learn from the sciences of complexity and chaos ( Bak, 1996; Briggs and Peat, 1989; Gleick, 1987; Poole and Van de Ven, 2004; Uhl-Bien and Marion, 2008), the only reasonable path involves contextualized, iterative, incremental actions to influence the intractable patterns of these issues over time. Problems that persist for educationalists in health professions meet these criteria and can be classified as “wicked” problems.

Intractable problems like these are pervasive and persistent across a range of health professions educational environments. If traditional problem-solving methods worked, one would expect such challenges to be less frequent, less widely recognized, and less permanent. Since they emerge in radically different settings and persist over time, it is clear that current methods are not sufficient to solve them. Individuals, teams, and institutions cope with such problematic patterns, but sustainable success does not come often. Professionals need some alternative approach to help them see, understand, and influence intractable problems in healthcare professions education.

## Wicked Problems

In the mid-1970s, Rittel and Webber ( Rittel and Webber, 1973) identified a new class of problems in social systems, which they called “wicked problems”. In the decades since, the concept of wicked problems has been expanded and articulated in a number of ways ( Zimmerman, Lindberg and Plsek, 2001; Weber, Lach and Steel, 2017). Three characteristics are common across diverse disciplines, theories, and stages of evolution of the concept. Wicked problems are:

1. Defined differently from different perspectives
2. Context dependent, but patterned across contexts
3. Impossible to solve completely

In practice, it is relatively easy to identify wicked issues. All the challenges defined by participants in our symposia (see Table 1) meet these three criteria. What is not so simple is to figure out how individuals and institutions should respond to wicked problems, given that they cannot be solved by traditional approaches. For this, we turn to the theory of complex adaptive systems (Dooley, 1996; Olson and Eoyang, 2001) and the practice of Human Systems Dynamics (G. Eoyang, 2011; G.H. Eoyang and Holladay, 2013).

## Human Systems Dynamcis (HSD)

Human Systems Dynamics (HSD) ( www.hsdinstitute.org) is a field of theory and practice derived from applications of complex adaptive systems to wicked problems in human environments. It provides an alternative to traditional problem “solving” approaches and supports professionals as they encounter otherwise intractable issues. The characteristics of wicked problems stymie the assumptions of traditional problem-solving methods, but HSD models and methods support innovative, adaptive, agile approaches that tame wicked problems, even when the problems cannot be solved.

HSD is built on the foundation of a simple, iterative cycle called Adaptive Action. Iterative cycles are widely accepted as a learning process ( Kolb, 1984). They are also recognized as a problem-solving and quality management method ( Baxley, Hubbs, Shay, and Karanian, 2011). HSD focuses on such a cycle to see, understand, and influence the complex patterns of systemic interactions that generate wicked problems and hold them in place.

The HSD version of a problem-solving and learning cycle consists of three questions: What? So what? and Now what? Adaptive Action works across scales because an individual, team, group, or institution can use the same questions. It also works across time scales (addressing cycle times of seconds to decades) and applies to multiple contexts (applying to personal, professional, and social issues). The three-step process and the familiar language make Adaptive Action accessible to a wide range of audiences and environments. It is particularly useful in response to wicked problems because it responds to the unique challenges they present. We now explore the connection between the three steps of the Adaptive Action cycle and the characteristics of wicked problems.

## Diverse Perspectives-What?

The first stage of Adaptive Action asks What? ( Eoyang and Holladay, 2013, p. 32). This step requires the user to pause and reflect on what IS. Too often, busy professionals speed through this question and move into action without fully understanding the nature of the problem. HSD uses a variety of methods to help groups uncover multiple views of the essential pattern of a wicked problem.

At the AMEE symposia, the idea of wicked problems and Adaptive Action were briefly introduced as were Open Space Technology, and self-organizing conversation ( Bates Evoy, 2016; Owen, 2008). We asked participants to identify wicked problems they wanted to focus on in small groups. People then organized themselves around the challenges that were most interesting to them. The first step of the exercise was to engage with wicked problems-What? Each person who was experiencing the same challenge was invited to share their views of the persistent patterns related to the group’s wicked problem.

In this short discussion, people realized that others shared the problem, but that each person saw the issue differently. The questions and insights came from different people around the circle with different roles, levels of experience, and personal perspectives. Each person added depth and complexity to the narrative of the wicked problem ( Ripley, 2018). By the end of this segment, individuals were beginning to appreciate others’ views and to create a more nuanced and tractable vision of their own wicked problem. In some ways, this step gives hope to those who suffer from wicked problems. They come to realize that they are not alone and to move beyond blaming (themselves and others) for their persistent problems. These difficult issues get normalized, and each person acknowledges the myriad ways that the same wicked problem can emerge.

All data collected was group data. No individuals were identified. How many groups, size of groups, length of workshop, follow-up data from participants’ What? So What? Now What? that we’re complete were used as examples.

## Context Dependent, but Patterned across Contexts - So what?

Even though wicked problems cannot be solved, they can be influenced. The purpose of this second stage of Adaptive Action-So what? ( Eoyang and Holladay, 2013, p. 64)-is to set conditions for individuals to imagine a wide range of options for action. Given the complex nature of the challenge, a wide range of steps could make a difference in the wicked, complex, problematic pattern. By sharing their experiences and observations, groups begin to generate new and innovative options for action. The purpose is to be and to feel less “stuck” in the wicked pattern.

One real challenge of wicked problems is that they are recognizable from one place or time to another, but they never appear in exactly the same way. Resistance to change, for example, is a pervasive problem, but no two people will feel or express their resistance in the same way. To make it even more complex, a single person is likely to express resistance in different ways at different times. During the So What? phase of Adaptive Action, people engage with their own contexts and constraints to recognize what is unique about the problem as they experience it and to explore innovative actions to leverage the opportunities and constraints that are unique to the challenge currently.

In Helsinki and Basel, groups were invited to spend 20 minutes considering the question, “So what are the opportunities and constraints related to this wicked problem?” The experienced varied greatly from one group to another. Some conversations were dominated by individuals who thought they had a solution for others. Other groups got stuck in What? and continued to reiterate their problem descriptions-focusing on constraints more than opportunities. Many groups, however, began to unlock possible pathways for action that could shift the wicked pattern, even though it would not solve the problem (See Table 2 for some sample actions). Given the wide range of quality among the conversations in Helsinki, in 2017, we realized that the groups could have used more detailed guidance and facilitation in the process. Consequently, In the 2018 symposium in Basel, we increased small-group time and improved the handouts to guide learning.

## Impossible to solve completely - Now what?

Solving problems is the ultimate goal in Western society ( Jullien, 2004). Even beyond a cultural preference, this pattern is central to the roles of the health professional and the educator. Expertise is the goal, and expertise is defined by the problems we can solve. We should not, therefore, be surprised when wicked, unsolvable problems become such barriers to effectiveness and efficiency in health professions education. The third step of Adaptive Action-Now what? ( Eoyang and Holladay, 2013, p. 82)-gives a disciplined alternative, when solution is not an option.

Our shared narratives often represent wicked problems as singular, coherent challenges. They are glorified as rocks in the road, walls to beat our heads against, or other kinds of immovable objects. Seen through the complex systems lens, with the aid of HSD models and methods, these wicked problems are converted into patterns. As opposed to problems, patterns exist at multiple scales. They have open edges and intricate structures within. Often, they can be seen to move on their own, without assistance from us. They are highly variable, so they offer a wide diversity of options for action.

When engaging with a problem as a pattern, the need for a solution evaporates. You cannot expect to change a whole complex pattern in one action. You have an alternative. Rather than removing the rock, one can merely shift the pattern, and it might become something altogether different. Or not! Complex adaptive systems are essentially unpredictable. There is no way to anticipate ahead of time whether an intervention will be a tipping point ( Bak, 1996; Gladwell, 2000) to shift the entire system or merely a drop of water in the ocean. Still, as responsible professionals, health care professions educationalists must take action.

The Now What? step of Adaptive Action invites reasonable and practical action in a given moment and situation, even with incomplete information. People are invited to do something--anything that is reasonable based on what they discovered in So what? We call this choice the “next wise action.” It may not be the perfect action, and it certainly won’t be the final action, but given what is known about the wicked problem-turned-pattern, it is the most wise action available. Physicians do this when they engage in presumptive treatment of a problem pending further evaluation.

One guideline is particularly important to effective Adaptive Action. Each situation is unique. That means that a next wise action for one person at one time in one context might not be so wise for someone else in another time and place. Each individual or group has to engage in its own Adaptive Action to find its own next wise action each and every time.

In their experience in the Open Space at AMEE in 2017 and 2018, individual participants saw and committed to new options for action. They had stated their wicked problems in similar ways, but because their contexts were so different, their wise actions were diverse. Some examples are included in Table 2.

**Table 2.** List of Proposed Actions (Now What?) for four wicked problems posed by participants at AMEE 2017 and 2018 Symposia

Provide education in a time-poor clinical situation	Prioritize predictable teaching activities Get feedback from the school for clinical teacher and student progress Need a metric--vacuum for feedback from academics Ask more questions to shift student thinking Structure and balance time of teaching and clinical practice Do a medical education course Look at literature for metrics Help students manage their learning
Teaching and learning “on the job” for junior medical staff	Start the conversation Understand how education is viewed by those you want to engage Understand what they need (e.g., feedback on good and what needs improvement) What do they need to feel appreciated and what that would look like? Simple thanks and formal recognitions Membership in a community of practice
Collaborative planning and implementation of curriculum changes	Ask the faculty what matters to them Share what matters to students and the institution Share research in teaching methodologies Value their contributions Create ownership of the curriculum development
Generating effective ethical practices for clinical and volunteer faculty	Provide supportive feedback Hugs to the few who are the exceptions Don’t want to lose them Ask them why they do it Find their motivations

Each of these proposed actions came from a participant, and no one committed to more than one next wise action. None of these actions will resolve the issue completely, but they will all make some kind of difference in the problematic pattern. Ultimately, even if the action does not work as planned, it will be an experiment that generates new understanding and options for the next Adaptive Action cycle.

The final point about the Now What? step of Adaptive Action is that it is never the end. Any action in a complex system brings unexpected consequences. That means that any “next wise action” will result in unpredictable system change, so every Now What? must be followed immediately with a next What? As each wise action generates consequences and impacts, the actor stays aware and adaptive to see what emerges (What?), to understand the changing patterns (So What?), and to choose the next, next wise action (Now What?).

The symposia sessions with participants was well received and useful for many. In retrospect, we would have made some design adjustments to accommodate the short, 90-minute session, the number and diversity of participants, and the expectations for applied learning. On the other hand, the session served as an experiment and a “next wise action” in building adaptive capacity for the complex field of health professions education.

## Conclusions

Health professions educators face an array of wicked challenges that emerge from the complexity of their expectations and environments. As long as they struggle to solve the unsolvable, they will experience frustration and failure. On the other hand, when we are able to distinguish tame, solvable problems from those that are wicked, we can access new methods of analysis and decision making that are fit for function in our complex, unpredictable, and fast-changing environments. Human systems dynamics offers one such pathway into effective action in complexity-Adaptive Action. The discipline of Adaptive Action guides individuals and groups to:

• What? Recognize the power of diverse perspectives when dealing with seemingly intractable issues
• So What? Acknowledge wicked problems as patterns, where each incident and each moment offer options for action
• Now What? Take a next wise action to shift the pattern and learn from the consequences, even when solutions are out of the question
• Continue the cycle of Adaptive Action to influence and tame wicked problems over time and contexts

## Recommendations

This experience with wicked problems of health professions educationalists has reinforced some of our underlying assumptions and challenged others. We recommend that the practice be continued and expanded to build adaptive capacity for individuals and systems engaged in complex educational settings around the world.

For future Symposia using Open Space technology with diverse groups of educationalists to explore wicked problems, we recommend:

• Dedicate less time to theory and overview of concepts and more in the practice of Adaptive Action to allow groups to explore their own Adaptive Actions more completely
• Provide more specific instructions and tighter constraints in terms of the use of time, the focus on conversations, and the expected output from the groups to help improve the specificity of the outcomes
• Facilitate individuals’ commitments to executing their next wise actions and sharing their learning with others
• Provide a method for participants to report their actions and findings following the meeting and share those reports with others

For the application of Adaptive Action more broadly across the field of health professions education, we recommend:

• Document and distribute case studies where Adaptive Action has been applied to wicked issues in health professions education
• Select one particularly wicked problem that is endemic to health professions education. Apply Adaptive Action to that issue in a variety of settings and track results and impacts over time
• Embed the concept of wicked problems and Adaptive Action into training and education for health professions educators to help them develop reasonable expectations for themselves and increased adaptive capacity in response to complex challenges they face.

## Take Home Messages

Wicked problems share three characteristics. They are: Defined differently from different perspectives; Context dependent, but patterned across contexts; Impossible to solve completely. Human Systems Dynamics is a field of theory and practice derived from applications of complex adaptive systems to wicked problems in human environments. The characteristics of wicked problems stymie the assumptions of traditional problem-solving methods. HSD models and methods support innovative, adaptive approaches that tame wicked problems. Adaptive Action is a simple, iterative cycle of three questions: What? So What? Now what? that works with wicked problems across time scales and multiple contexts.

## Notes On Contributors

Glenda H. Eoyang, PhD is Founding Executive Director of the Human Systems Dynamics Institute. Circle Pines, MN US.

Stewart Mennin, PhD is a Consulting Associate at the Human Systems Dynamics Institute, Adjunct Professor, Department of Medicine, Uniformed Services Institute of the Health Sciences, Bethesda, Md., Professor Emeritus, Department Cell Biology & Physiology and former Dean for Educational Development and Research, University of New Mexico, School of Medicine, Albuquerque, NM USA. He is the author and creator of ESME, Essential Skills in Medical Education and ESIA, Essential Skills in Action.

## Appendices

Appendix A lists participants at the AMEE Symposia on Wicked Issues held in Helsinki, 2017 and Bern, 2018, who contributed to the work represented in this manuscript and who gave permission to the authors to have their names appear in this manuscript.

**Appendix A:**

Name	Name
Aphang, Meylin	Nevalainen, Maarit
Arikoski, Pekka	Noehr, Suzanne
Bullock, Shane	Norris, Madeleine
Chang, Jason	Nowakowski, Michael
Cheng, Nicholas	Parish, Emma
Chue, Shien	Petroni Mennin, Regina
Deshparde, Saee	Pippa-Savolainen, Eija
Dimitriades, Konstantinos	Pope, Lindsey
Ditchfield, Carol	Prevost, Yolanda
Edgren, Gudrun	Raatikainen, Kaisa
Gray, Amy	Rcsiweth, Anant
Gummesson, Christina	Roder, Carrie
Hammond, Jennifer	Rogers, Kem
Heinaaho, Emil	Rundlof, Anna-klara
Helin-Salmivaara, Arja	Schlegel, Elisabeth
Hevenn, Maura	Seddon, Kate
Heyligers, Ide	Smallwood, David
Hultgren, Catharina	Sornalingam, Sangeetra
Irish, Bill	Sum, Sutida
Jason, Hill	Tan, Brian
Jones, Liz (Linda)	Tan, Chee-kiat
Klitgaard, Tine	Tobin, Hannah
Krishnasamy, Charmaine	Tsai, Jer-Chia
Lahtinen, Alexandra	Tun, SanYuMay
Lai, Christine	van Dam, Marjel
Launio, Sara	van Woozile, Tamare
Luciom Machado, Jose	Watter, Maria
Ludman, Ronald	Weissman, Gaby
Luginbuehl, Helena	Westberg, Jane
McCrory, Richard	Woon Li, Seo
Metsavainio, Kirsimarja	Wozniak, Helena
Mier, Maria	Yee, Look
Myers, Junette	Yeh, Huei-Ming
