# Supplementary material for: Wicked Problems in Health Professions Education: Adaptive Action in Action
Source: MedEdPublish (2016). 2019 Dec 10;8:226. [Version 1] doi: 10.15694/mep.2019.000226.1 (PMC10712497; doi:10.15694/mep.2019.000226.1)
Supplement: Peer review history

# Reviewer report: Reviewer response for version 1

**DOI:** 10.21956/mep.19934.r30241

**Published:** 2020-01-18

**Authors:** Joachim Sturmberg

**Affiliations:** 1 University of Newcastle, Australia

This review has been migrated. The reviewer awarded 4 stars out of 5 This article is a succinct description of a particular approach to managing wicked problems in the education sector. Not being – principally – an educationalist, I can clearly see the potential of this method in managing an – ? isolated – wicked problem. As an academic I share some of Tom Dornan’s observations and would like to add another, namely the “system wide” perspective. A wicked problem, as described here, represents a phenotypical expression of a particular system’s behaviour. Hence it is important to also map the system structure and connections that contribute to its dynamics, only then will one reach an overall appreciation of the problem in hand. This overall appreciation, I feel, will not only help to understand the issue more deeply but also find the “wisest” possible next step in managing the problem.

Reviewer Expertise:

NA

No decision status is available

# Reviewer report: Reviewer response for version 1

**DOI:** 10.21956/mep.19934.r30245

**Published:** 2020-01-10

**Authors:** Tim Dornan

**Affiliations:** 1 Queen's University Belfast

This review has been migrated. The reviewer awarded 3 stars out of 5 This article draws a very important topic to readers’ attention: a tendency for people at the present time to represent educational problems as very tangible ones (‘incompetence’, ‘unsafe behaviour’, ‘unprofessionalism’, ‘inefficiency’) and place undue emphasis on applying predetermined solutions to remediate ‘perpetrators’ of these problems. The popularity of competence-based education, which focuses strongly on assessing individual students’ or doctors’ competence and bringing it up to acceptable levels, epitomises this. Enthusiasm for applying stock solutions to stock problems, which is widespread in contemporary society, has been termed ‘Fordism’ after Henry Ford, who pioneered a production line approach to manufacturing. The authors of this article are telling us that, rather than requiring production line methods, social problems tend to be complex and require craft rather than automation. I agree. The authors offer useful rules of thumb for the craft of tackling complex social problems.I commend to readers’ attention some points made by the authors: that it is important to examine and explore complex problems carefully before attempting to solve them; that the imaginative development and evaluation of candidate solutions is a constructive approach to problems; and that taking an incremental approach is better than aiming to solve problems in one go.As a post-publication reviewer, though, I think it is important also to provide a critical counterpoint to the authors’ narrative. There are some general features of the article that encourage scholarly criticism. For example:• The narrative is descriptive and enthusiastic rather than critical; this should prompt caution• It tends to make sweeping generalisation: ‘solving goals is the ultimate goal in Western Society’ (who’s ultimate goal?); ‘expertise is the goal’ (who’s goal?). We should be careful, then, not to be yield too willingly to the authors’ enticing advocacy for their approachThere is one specific feature of the article, which I feel particularly calls for critical comment: the authors are both affiliated to a ‘Human Systems Dynamics Institute’. I know nothing about the Institute and have no idea whether or not the authors have competing interests, nor am I particularly interested. As an academic critic, though, I am a bit bothered by the authors offering ‘principles and practices from human systems dynamics’ as a healing balm for the ills of our age. Specifically, the authors imply that human systems dynamics offers novel and unique insights. In reality, the principles and practices the authors espouse date back to the creative energy of Kurt Lewin in the middle third of the twentieth century, if not earlier. Quality improvement (QI), action research, and other cyclical improvement methods are based on just the same approach, even if they use a different language from the one the authors use. The one contemporary approach that, for me, adds real value to Lewin’s work is Engeström’s suite of ‘formative interventions’, based on Activity Theory. To conclude, I find the notion of ‘wicked problems’ relevant and helpful, even if presented in too catchy a way for my sceptical academic comfort. I support the authors’ general approach and hope readers will be motivated to persevere in tackling problems that don’t yield to their first improvement efforts. I urge readers, though, to see the central messages in all their simplicity rather than regard Human Systems Dynamics as ‘the’ approach. Action research has been described as a signature methodology for education research. Let’s give Kurt Lewin, who first thought of it, credit for his relentless pursuit of social justice. Let’s be inspired by Eeyong and Mennin to continue Lewin’s mission in the present age. For those who want genuinely new ideas, I commend the work of Engeström.Tim Dornan

Reviewer Expertise:

NA

No decision status is available

# Reviewer report: Reviewer response for version 1

**DOI:** 10.21956/mep.19934.r30242

**Published:** 2019-12-27

**Authors:** Susan Van Schalkwyk

**Affiliations:** 1 Stellenbosch University

This review has been migrated. The reviewer awarded 5 stars out of 5 I agree with the previous reviewers that this is really an excellent article. I am familiar with human systems dynamics and the adaptive action process, but found this well-described and practical application most useful. The notion of problems not having solutions potentially frees both students and educators from the burden of always having the answer. It gives us license to essentially ‘sit with’ challenges (focus on the ‘what’ and the ‘so-what’) before rushing into ‘now what’. These are important ideas. It was also great to read about the outcomes from the two AMEE symposia emphasizing the value of this type of engagement at conferences to give ‘voice’ to a large group and to build shared understandings (look for patterns), among a wide range of health professions educationalists.

Reviewer Expertise:

NA

No decision status is available

# Reviewer report: Reviewer response for version 1

**DOI:** 10.21956/mep.19934.r30244

**Published:** 2019-12-22

**Authors:** Alan Bleakley

**Affiliations:** 1 University of Plymouth

This review has been migrated. The reviewer awarded 5 stars out of 5 This is a terrific article and such a privilege to review. The authors frame insoluble contradictions in healthcare education as ‘wicked problems’. Where we frame issues as problems we search for solutions, but wicked problems demand another mindset – first, one of appreciation (pattern recognition); and second, of “wise action” (contextualized, probably partial, but innovative). Contradictions usually arise from the concrete reality of complex and turbulent practice presentations meeting the idealism of approaches such as competence-based learning that stress standardization such as uniform outcomes. As I read the clear and expertly framed Abstract I thought of the tensions between ‘smooth/ de-territorialized’ and ‘striated/ territorialized’ phenomena articulated by Deleuze and Guattari. The authors are experts in the field of complexity thinking applied to healthcare education and practice. Here, they remind us that ‘problem solving’ is an inappropriate strategy for facing wicked problems. They also point out that while intractable problems do not invite solutions, they do present as patterns that can be appreciated, understood and influenced. This is best achieved through working knowledge of complex adaptive systems theory and systems dynamic practice. The paper presents results of a collaborative dialogue across a large number of informed health professionals bringing a range of wicked problems identified within their communities of learning and practice. The most frequently identified complex issue was “resistance to change”. (This has been identified in Yrjö Engeström’s work as regression to tradition or ‘will-to-stability’, rather than progression to innovation as ‘possibility knowledge’ or learning-for-change, where an individual locus of expertise shifts to a collective locus of expertise). The authors list 23 such wicked problems that, in both Activity Theory and Actor-Network Theory, would be termed ‘contradictions’ and would be seen as sources of potentially productive change rather than regressive frustration and crystallization. Such wicked problems could potentially expand activity systems or learning frames as long as the mindsets of participants also changed to embrace their structures - defined by complexity and open (human) systems dynamics. This means defying reductionism to embrace the artistry of learning and practice, and adopting incremental and iterative activity that is contextualized and multi-faceted. The authors again make a persuasive case for adopting sensemaking that embraces intractable problems rather than using reductionist approaches. We might compare this with Nietzsche’s ‘philosophizing with a hammer’. By this Nietzsche did not mean just hitting hard, but rather choosing the right tool for the job – the authors, from their own innovative modeling – stress “adaptive” and “agile” approaches to “tame” wicked problems. (I have a bit of an issue with “tame” – isn’t the approach the authors describe more one of ‘alignment’ and ‘appreciation’, developing adaptive actions that are at once appreciative and active?) Through a ‘what?’, ‘so what?’ and ‘now what’ cycle, participants engage at a deep level with wicked problems. This turns events into experiences, and is beautifully and clearly described – a joy to read!P4 I am a bit of a stickler concerning ‘data’ as plural (‘datum’ singular). Thus, “all data collected were group data” (not “was”).

Reviewer Expertise:

NA

No decision status is available

# Reviewer report: Reviewer response for version 1

**DOI:** 10.21956/mep.19934.r30243

**Published:** 2019-12-10

**Authors:** Felix Silwimba

**Affiliations:** 1 University of Lusaka

This review has been migrated. The reviewer awarded 5 stars out of 5 this conference report has made me aware of issues have questioned concerning the conduct of lecturing staff and students in university. I now appreciate wicked issues. it is a very educative write up.

Reviewer Expertise:

NA

No decision status is available
